# Independent neural circuits encode the dynamics of social interaction in rats

**DOI:** 10.1371/journal.pbio.3002396

**Published:** 2023-11-30

**Authors:** Natália Madeira, Cristina Márquez

**Affiliations:** 1 CNC-UC—Center for Neuroscience and Cell Biology, University of Coimbra, Coimbra, Portugal; 2 CIBB—Centre for Innovative Biomedicine and Biotechnology, University of Coimbra, Coimbra, Portugal

## Abstract

This Primer explores a study in PLOS Biology which demonstrates a key role of the central amygdala and specific circuits projecting to and from this brain area in the initiation versus maintenance of positive social interactions.

## Teaser

A study in *PLOS Biology* by Rojek-Sito and colleagues demonstrates a key role of the central amygdala and specific circuits projecting to and from this brain area in the initiation versus maintenance of positive social interactions.

Social interactions shape our daily lives and the way we learn about the world, having a deep impact on our emotions and physiology. The need to seek social contact and form relationships is a strong motivator, not only for humans but for many other social species. Despite its importance, the neural circuits underlying how the brain initiates and engages in social interactions are still poorly understood.

In this volume of *PLOS Biology*, Rojek-Sito and colleagues [1] address a very interesting and important question: How different facets of a dynamic social interaction are instantiated at the level of multiple neural circuits? To explore this, they perform a very elegant and detailed series of gain- and loss-of-function experiments, by using opto- and chemogenetic approaches in specific brain circuits in rats, which allow them to identify different networks that are involved in each of these processes.

Social interactions are dynamic events. The initiation and maintenance of a social encounter are 2 distinct stages critical for the development of successful social interactions. They are functionally different, and both determine the animals’ ability to connect with others. For an initial interaction to occur, it requires a motivation to approach other individuals, processes related to social recognition and interpretation of social cues. Then, the ability to maintain social contact will rely on the behavioral attunement with the interacting partner, reciprocation of social actions, and further evaluation of others’ social behaviors and emotional states. However, until now, these processes have not been independently addressed, as more coarse-grained measures of social investigation have been generally favored in the field. To our knowledge, this is the first attempt to disentangle the neural circuits behind the initiation versus the maintenance of a social interaction.

In this manuscript, Rojek-Sito and colleagues start by demonstrating the important role of the central amygdala (CeA) in highly motivated social interactions, i.e., after social isolation. Using a combination of neuroanatomy and optogenetic tagging and reactivation of behaviorally salient cells, they prove the existence of “social cells” in CeA. These cells are active during social interactions and when optogenetically reactivated, they increase the motivation to interact with conspecifics. Interestingly, CeA social cells are not coding for general reward or motivation, as they are specific for social interaction and not active, for example, during food-seeking tasks. Previous reports have related other parts of the amygdala with social behaviors, especially the medial amygdala [2] or basolateral amygdala [3]. The evidence that relates to the CeA in social cognition is scarce, with notable exceptions [4,5], and the present work reinforces the importance of the CeA in coding the value and categorization of a rewarding event.

In agreement with recent studies in other brain areas [6,7], the existence of an overlap between cells active for social or food rewards was not complete. Furthermore, the functional study of the projections coming from and projecting to CeA, and the neurochemical profiles obtained after social or food reward situations, revealed that the circuits were specifically involved in social events and independently encoding the initiation versus maintenance aspects of a social interaction.

Using viral strategies to chemogenetically silence specific neural circuits in a sequential manner, Rojek-Sito and colleagues show that dopamine (DA) neurons in the ventral tegmental area (VTA) are crucial for the initiation of a social interaction. The VTA is one of the core nodes of the brain’s reward system and is known to drive motivated behaviors and process reward information. The involvement of the VTA in motivation for social interactions had been previously described [8,9]. The present study expands these initial findings and relates DA function in the VTA to the motivation to initiate an interaction through its projections to cortical areas (anterior cingulate cortex (ACC) and orbitofrontal cortex (OFC)). DA neurons in the VTA projecting to both ACC and OFC, and the projection from OFC to the CeA, are necessary for the initiation of a social interaction but not required for its maintenance.

On the other hand, the maintenance of social interactions requires the activity of the neurons from ACC that project to CeA and from there to the VTA. Although the study does not provide the temporal resolution to ascertain which areas are first activated, it is tempting to think that top-down control of DA cells after the initiation of an interaction mediates how social interactions are reinforced to promote the reciprocation of the social cues from others and thus continue engaging in the interaction. Future experiments should aim to provide finer temporal resolution to these interesting findings. Simultaneous neural recordings in the different brain areas identified would help to clarify whether there is a sequential and/or temporal pattern in the recruitment of these circuits during the initiation and maintenance of social interaction.

Social interactions are the product of concerted neural activity in different brain regions. The present manuscript escapes from the tendency of one circuit–one behavior and embraces the complexity that underlies a simple social interaction. By starting their quest on the role of CeA, they find different brain areas in the cortex and midbrain that are differentially engaged in the dynamics of social interaction. This does not preclude that other brain regions might be also involved in these processes. For example, the authors highlighted the possible role of hypothalamic regions in the control of CeA activity in social interactions, maybe through its connections from the medial amygdala [2]. Future studies will complement the findings exposed in this elegant work.

In conclusion, this study provides the first evidence that distinct neural circuitries mediate different aspects of dynamic social interactions. This is a very important discovery that opens the door to interesting new questions. The distinction of 2 multicircuit networks differently controlling the initiation versus the maintenance of social interactions is paving the way for new studies. For example, these novel findings might help to better understand different animal models of psychiatric disease, with prominent social dysfunction characteristics, which might be differently affected in these specific circuits.

**Fig 1:**
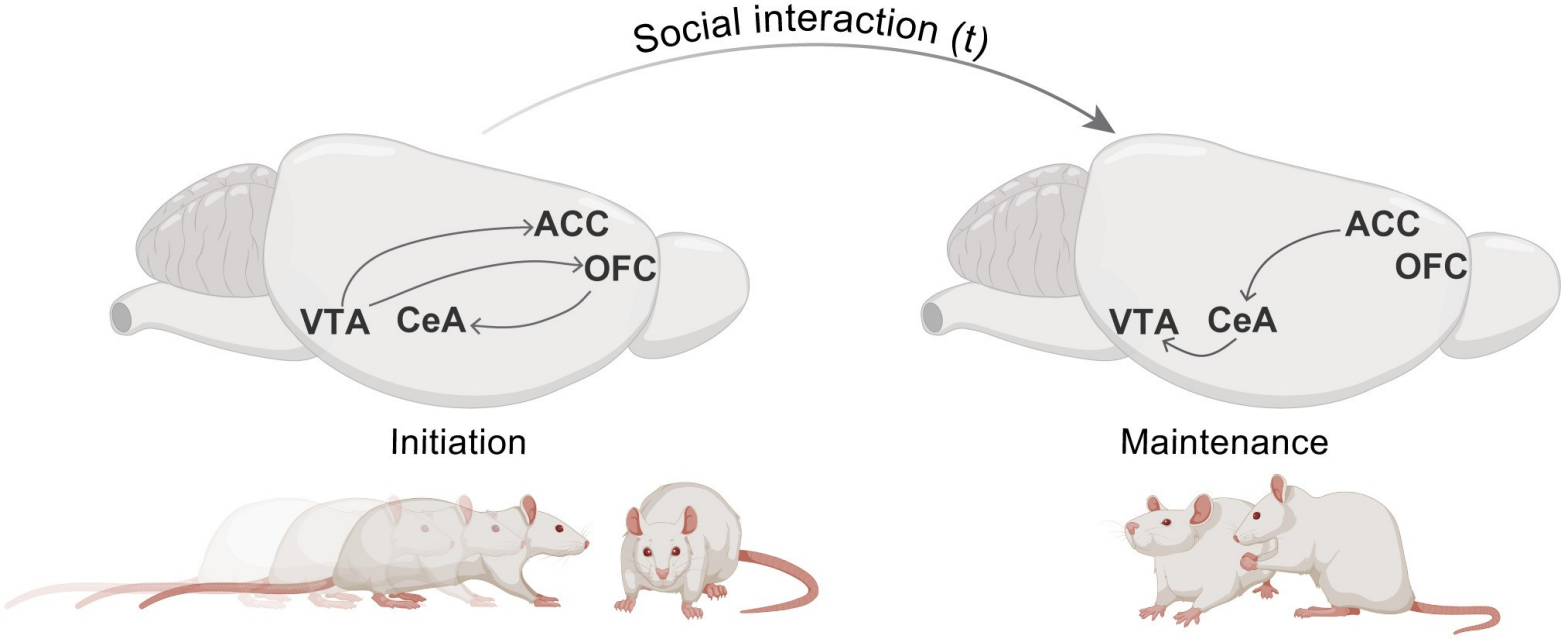
Independent circuits are involved in the initiation versus maintenance of social interactions. The initiation of social interactions is marked by an active motivation to approach conspecifics. This behavior is characterized by the involvement of the projections from VTA to ACC, VTA to OFC, and from OFC to CeA. The maintenance of social interaction is mediated by a circuit that involves projections from the ACC to CeA and from CeA to VTA, allowing the animals to maintain social contact with the interacting partner. ACC, anterior cingulate cortex; CeA, central amygdala; OFC, orbitofrontal cortex; VTA, ventral tegmental area.

## Abbreviations

ACC: anterior cingulate cortex
CeA: central amygdala
DA: dopamine
OFC: orbitofrontal cortex
VTA: ventral tegmental area
